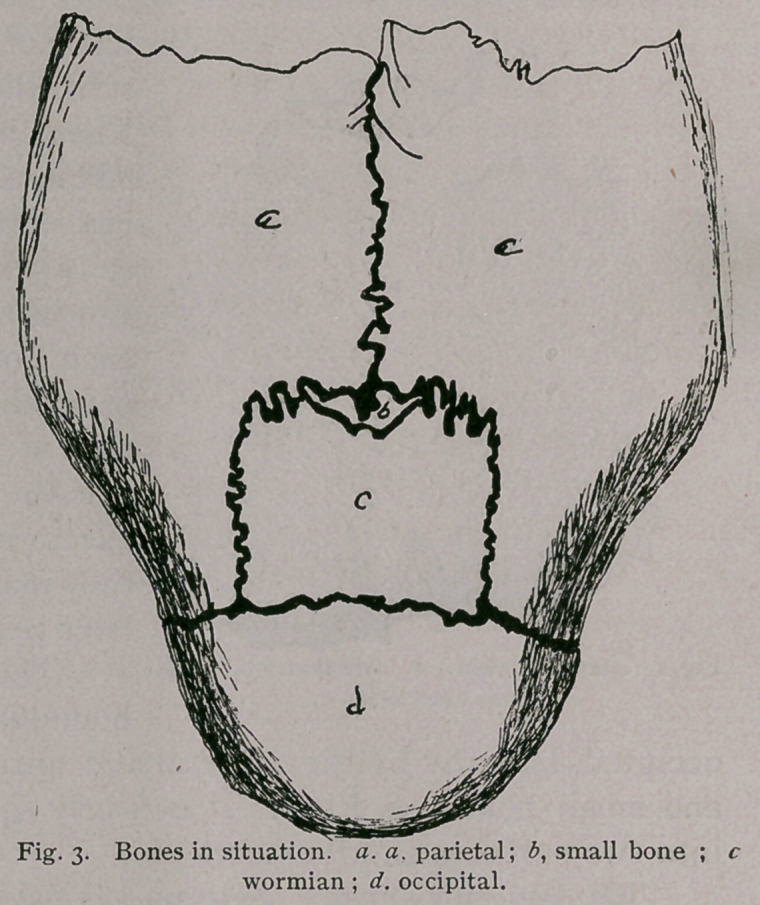# The Wormian and —?— Bones1Hinebauchian bone (Ed).

**Published:** 1890-11

**Authors:** T. D. Hinebauch

**Affiliations:** Pardue Experiment Station, Lafayette, Ind.


### THE JOURNAL
OF
### COMPARATIVE MEDICINE AND VETERINARY ARCHIVES.

VOL. XI. NOVEMBER, 1890. NO. 11.

### THE WORMIAN AND ——?——[1] BONES.

BY T. D. HINEBAUCH.
Pardue Experiment Station, Lafayette, Ind.

IN examining the bones of the head of young colts from birth and four to six weeks of age, I have particularly noticed the presence of a wormian and another bone; the latter, which has not heretofore been described as far as I can learn. These bones were found present in all such animals that I have taken the pains to critically examine. As age advances these bones unite with each other and with the parietal on either side, so that at one to three years of age they lose their identity, except in rare instances where they may be traced at a much later period.

Fig. 1. Superior view. A, wormian; *a*, small bone anteriorly.

The wormian bone is bounded posteriorly by the occipital, laterally by the parietal and anteriorly by the parietal and small bones, *a*, Fig. 1. It is nearly square and divides itself into two surfaces and four borders.

The superior surface (external) is flat and roughened. The internal surface is flat and smooth along its lateral borders.

[1]Hinebauchian bone (Ed).

On the mesian line there is a spur-like process, *c*, Fig. 2, the ossific tentorium projecting downward and backward. The ossific tentorium becomes wider from before back near its apex. It presents two surfaces, two borders and an apex. The anterior sur face is con cave and thick superiorly, convex and thin inferiorly. The posterior surface is concave, and presents a projection at its upper third, which is directed backward and slightly upward. The apex is rounded and subdivided. The anterior border has a triangular notch, which receives the bone *a*, Fig. 1. The lateral borders are straight and sutured to receive the sutures of the parietal bones. The posterior border is thick, straight and somewhat beveled from above downward and backward. It is also sutured and articulates with the occipital.

Fig. 2. Lateral view. B, wormian; *b*; small bone; *c*, ossific tentorium; *d*, inferior extremity of small bone.

Anterior to the wormian bone is a small, irregular-shaped bone, which closely fits in its anterior border and fills up the space between it anteriorly and the parietal bone, *b*, Fig. 3.

Fig. 3. Bones in situation. *a. a.* parietal; *b*, small bone; *c* wormian; *d*. occipital.

This bone may be divided into two surfaces, two borders and two extremities. The anterior surface is straight and thick superiorly, concave and thin inferiorly. The posterior surface is concave and notched superiorly, convex and thin inferiorly. The lateral borders are smooth and straight and marked throughout their length by a ridge.

The superior extremity is irregular in outlines and rough, the inferior sharp and projecting.

## Figures and Tables

**Fig. 1. f1:**
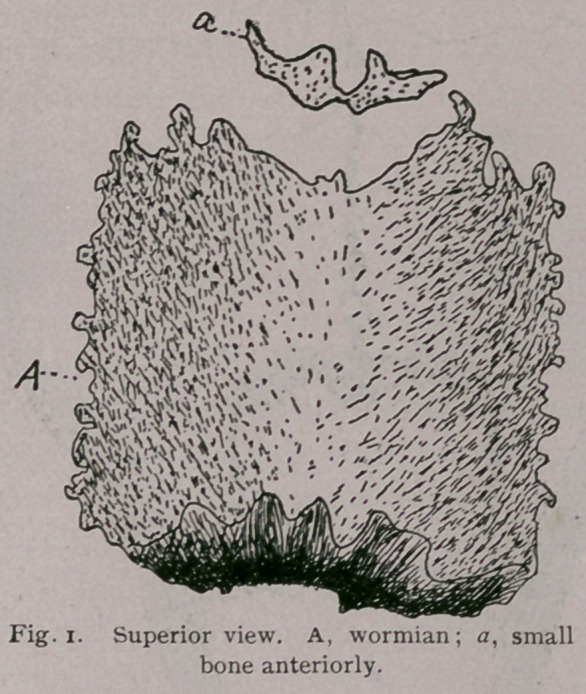


**Fig. 2. f2:**
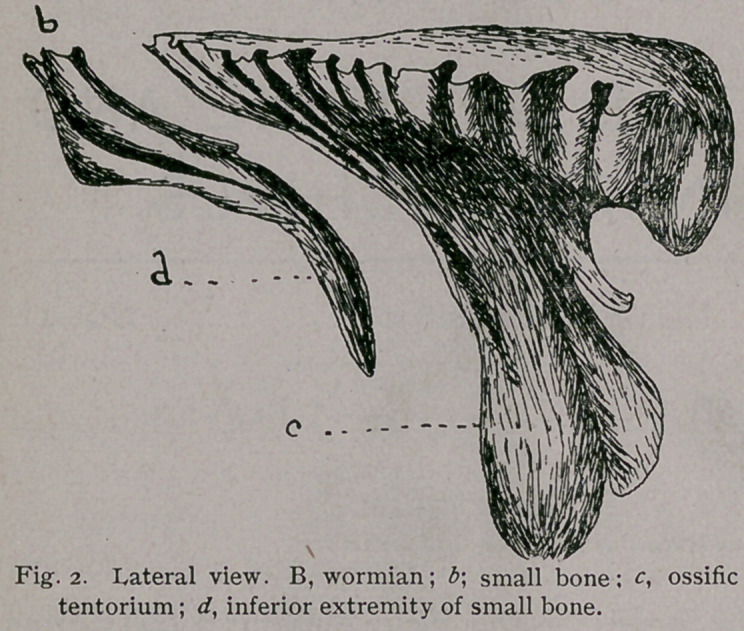


**Fig. 3. f3:**